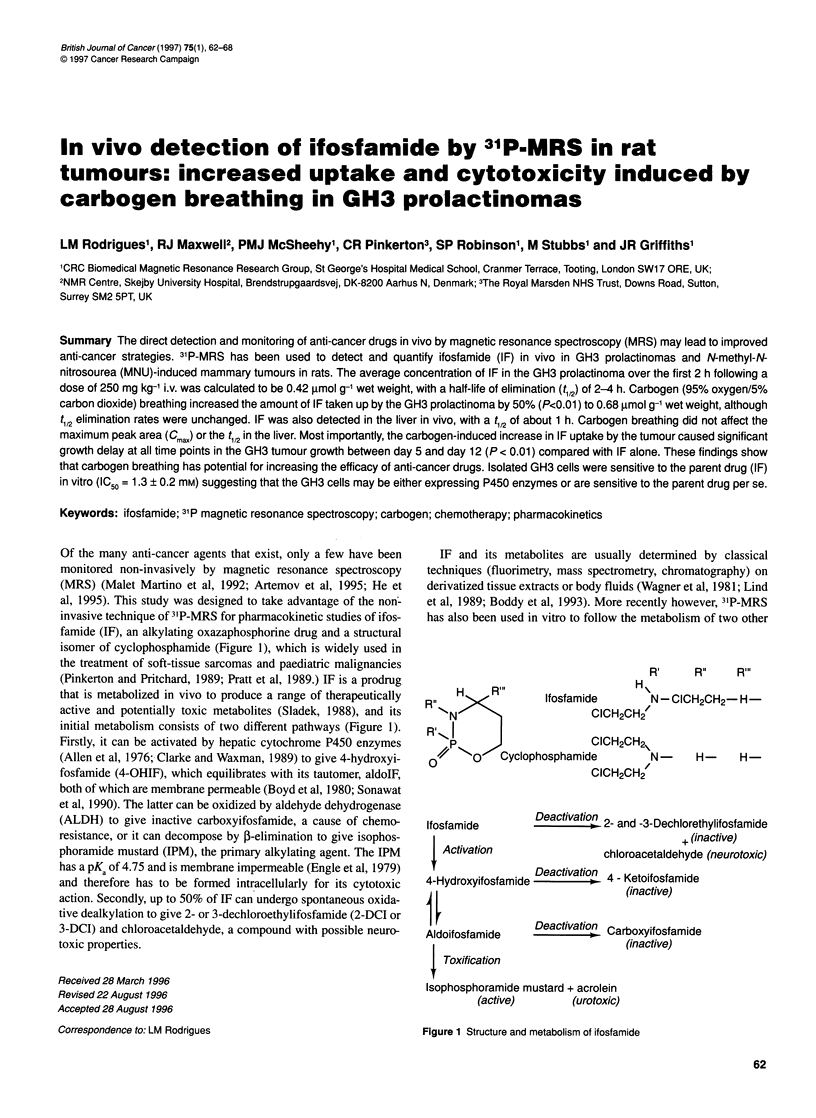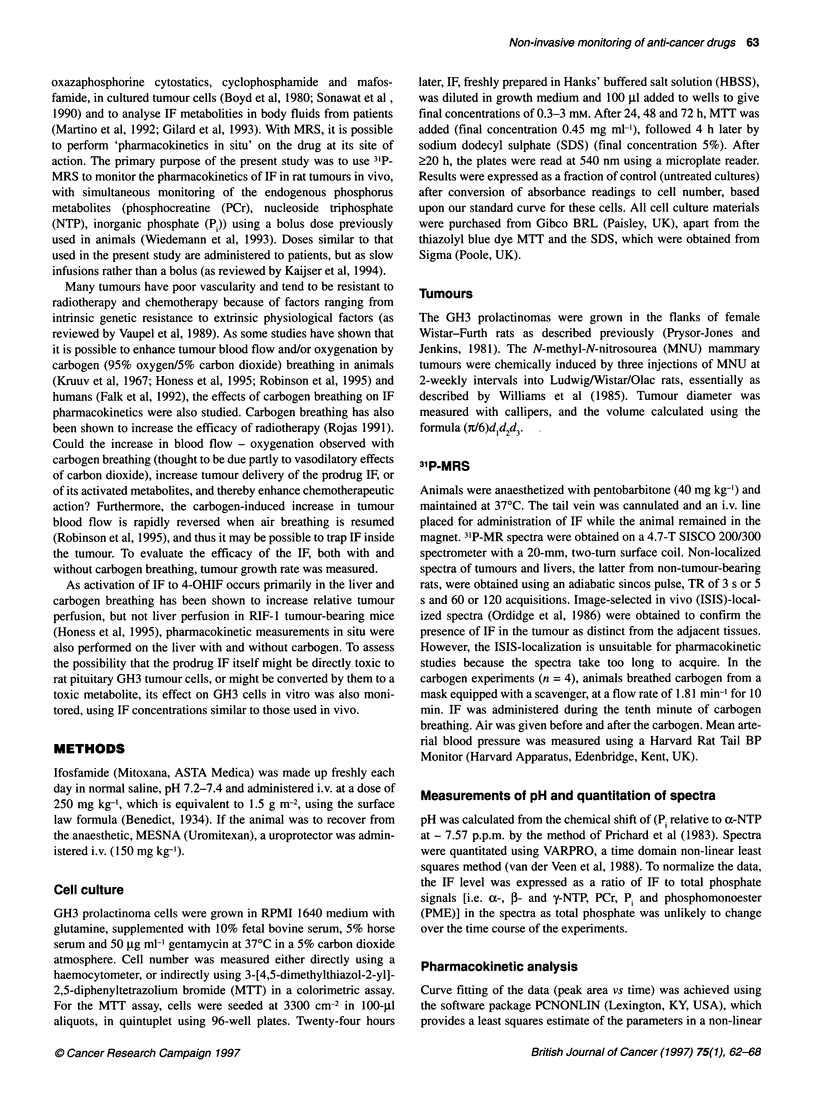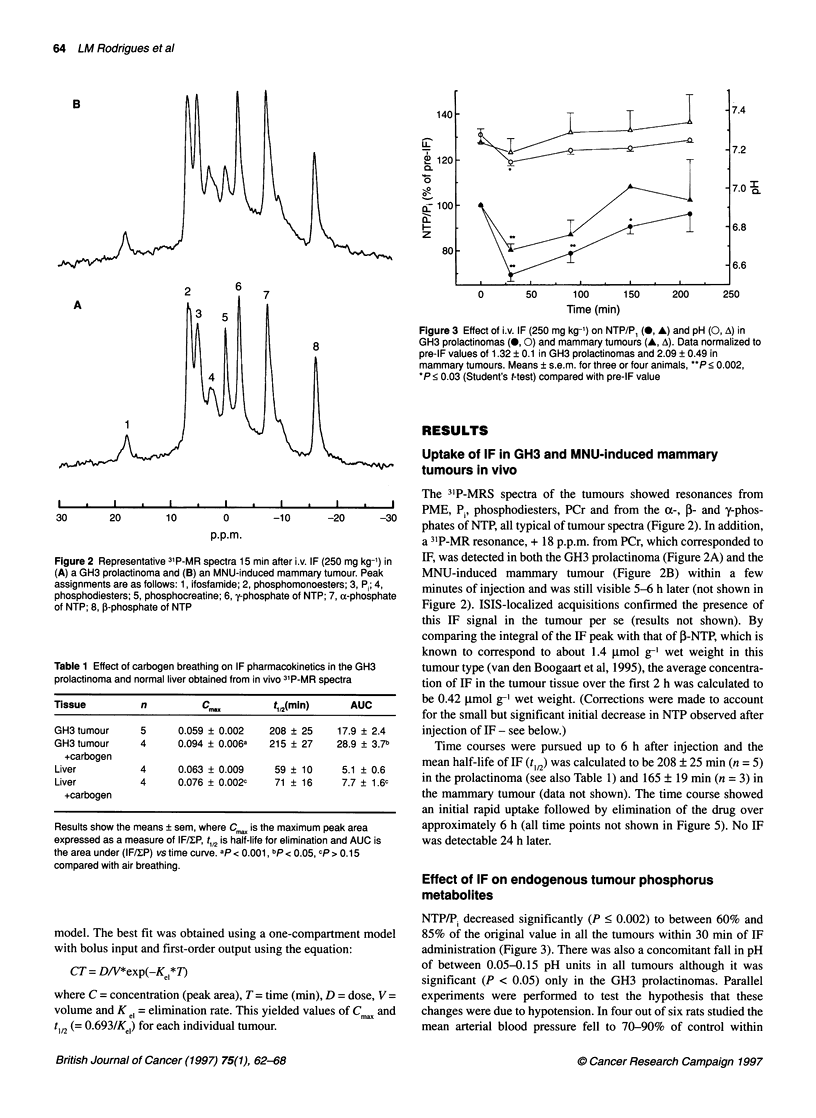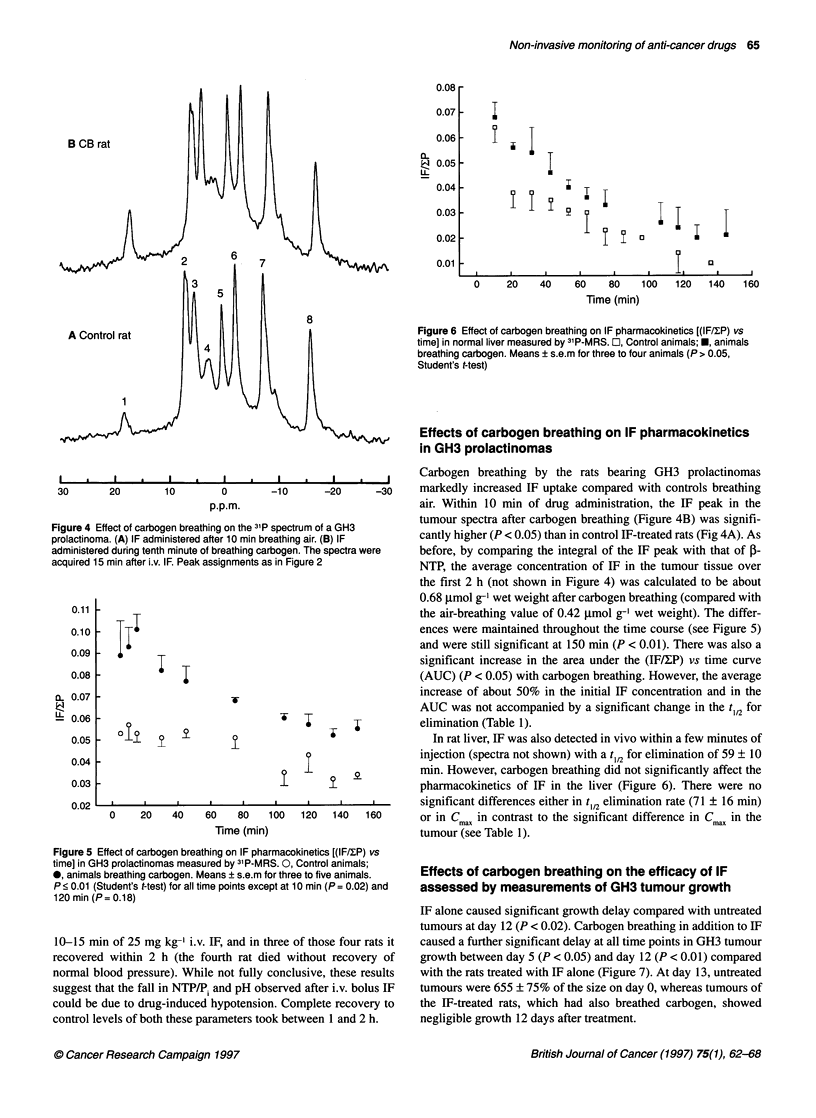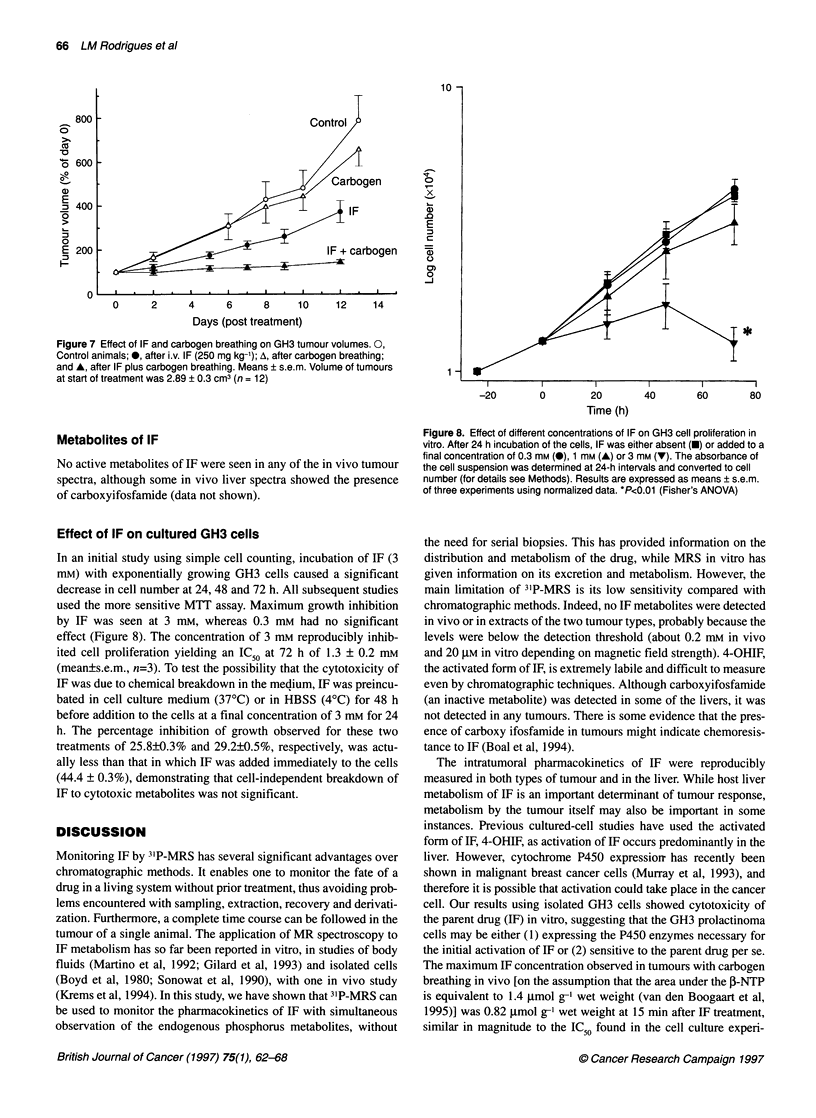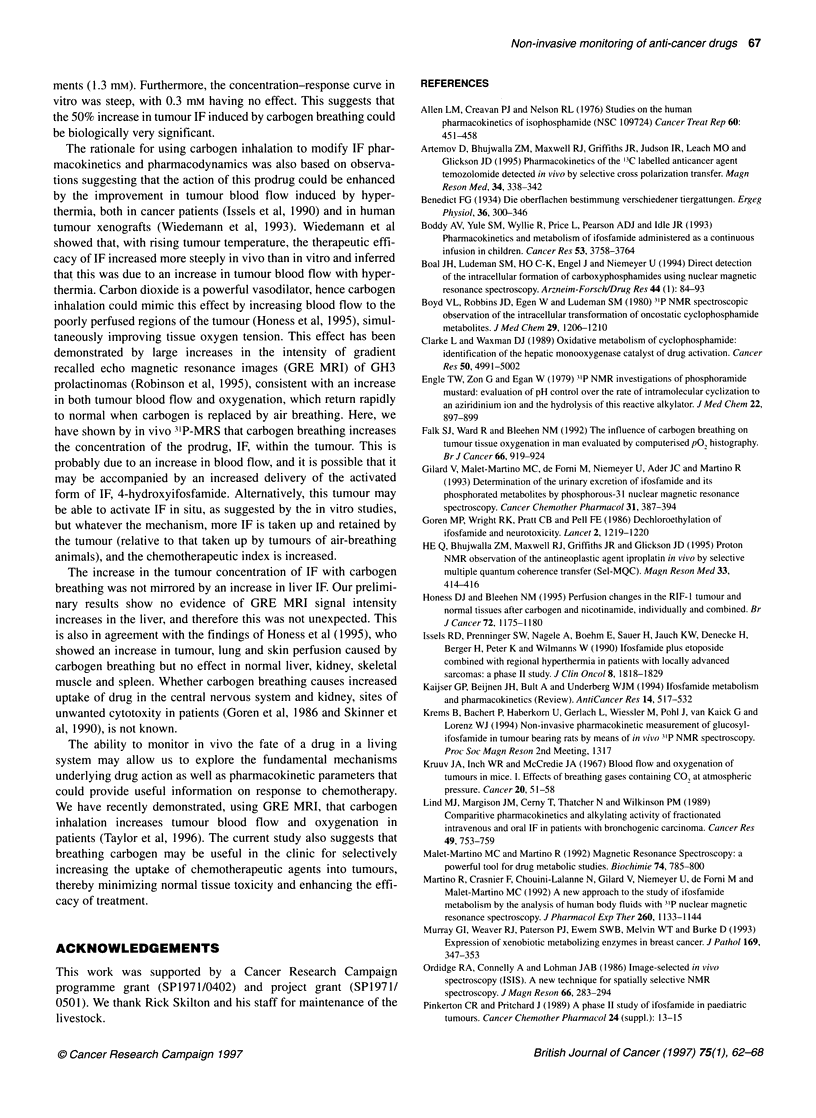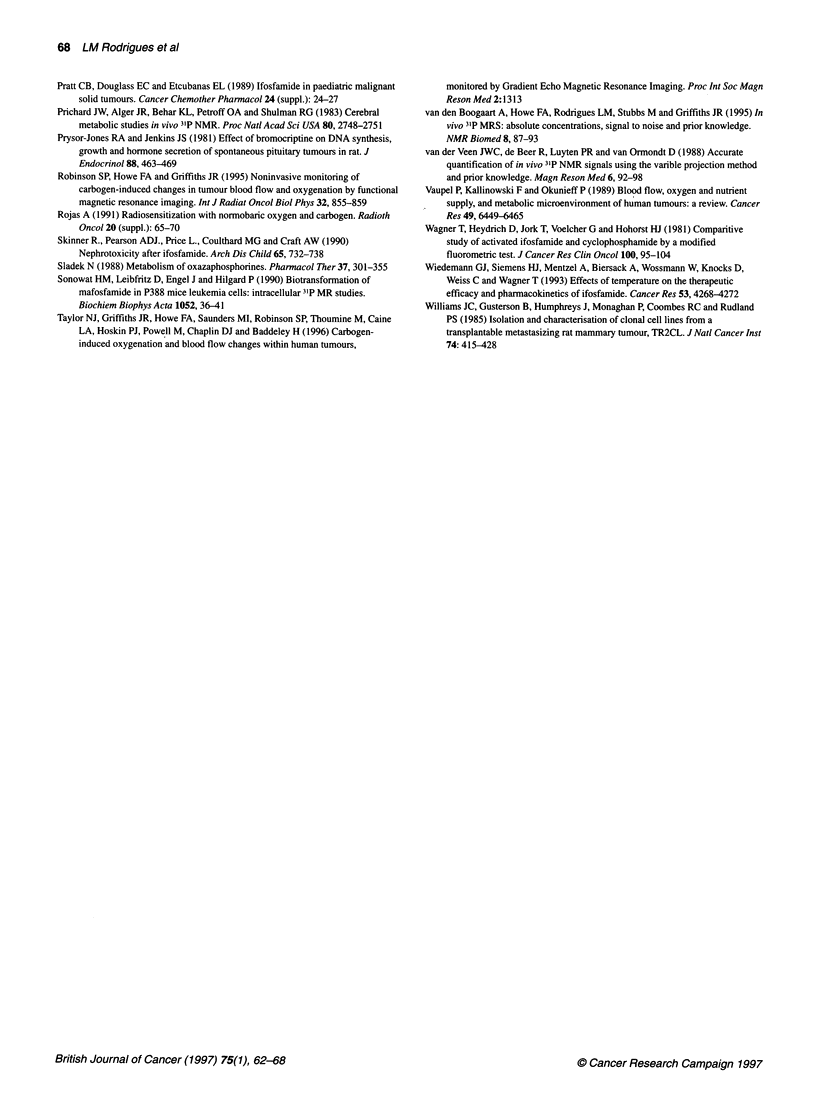# In vivo detection of ifosfamide by 31P-MRS in rat tumours: increased uptake and cytotoxicity induced by carbogen breathing in GH3 prolactinomas.

**DOI:** 10.1038/bjc.1997.10

**Published:** 1997

**Authors:** L. M. Rodrigues, R. J. Maxwell, P. M. McSheehy, C. R. Pinkerton, S. P. Robinson, M. Stubbs, J. R. Griffiths

**Affiliations:** CRC Biomedical Magnetic Resonance Research Group, St George's Hospital Medical School, London, UK.

## Abstract

The direct detection and monitoring of anti-cancer drugs in vivo by magnetic resonance spectroscopy (MRS) may lead to improved anti-cancer strategies. 31P-MRS has been used to detect and quantify ifosfamide (IF) in vivo in GH3 prolactinomas and N-methyl-N-nitrosourea (MNU)-induced mammary tumours in rats. The average concentration of IF in the GH3 prolactinoma over the first 2 h following a dose of 250 mg kg-1 i.v. was calculated to be 0.42 micromol g-1 wet weight, with a half-life of elimination (t1/2) of 2-4 h. Carbogen (95% oxygen/5% carbon dioxide) breathing increased the amount of IF taken up by the GH3 prolactinoma by 50% (P<0.01) to 0.68 micromol g-1 wet weight, although t1/2 elimination rates were unchanged. IF was also detected in the liver in vivo, with a t1/2 of about 1 h. Carbogen breathing did not affect the maximum peak area (Cmax) or the t1/2 in the liver. Most importantly, the carbogen-induced increase in IF uptake by the tumour caused significant growth delay at all time points in the GH3 tumour growth between day 5 and day 12 (P< 0.01) compared with IF alone. These findings show that carbogen breathing has potential for increasing the efficacy of anti-cancer drugs. Isolated GH3 cells were sensitive to the parent drug (IF) in vitro (IC50 = 1.3 +/- 0.2 mM) suggesting that the GH3 cells may be either expressing P450 enzymes or are sensitive to the parent drug per se.


British Journal of Cancer (1997) 75(1), 62–68
© 1997 Cancer Research Campaign

### In vivo detection of ifosfamide by $^{31}$P-MRS in rat tumours: increased uptake and cytotoxicity induced by carbogen breathing in GH3 prolactinomas

**LM Rodrigues[1], RJ Maxwell[2], PMJ McSheehy[1], CR Pinkerton[3], SP Robinson[1], M Stubbs[1] and JR Griffiths[1]**

[1]CRC Biomedical Magnetic Resonance Research Group, St George's Hospital Medical School, Cranmer Terrace, Tooting, London SW17 ORE, UK; [2]NMR Centre, Skejby University Hospital, Brendstrupgaardsvej, DK-8200 Aarhus N, Denmark; [3]The Royal Marsden NHS Trust, Downs Road, Sutton, Surrey SM2 5PT, UK

**Summary** The direct detection and monitoring of anti-cancer drugs in vivo by magnetic resonance spectroscopy (MRS) may lead to improved anti-cancer strategies. $^{31}$P-MRS has been used to detect and quantify ifosfamide (IF) in vivo in GH3 prolactinomas and *N*-methyl-*N*-nitrosourea (MNU)-induced mammary tumours in rats. The average concentration of IF in the GH3 prolactinoma over the first 2 h following a dose of 250 mg kg$^{-1}$ i.v. was calculated to be 0.42 μmol g$^{-1}$ wet weight, with a half-life of elimination ($t_{1/2}$) of 2–4 h. Carbogen (95% oxygen/5% carbon dioxide) breathing increased the amount of IF taken up by the GH3 prolactinoma by 50% ($P$<0.01) to 0.68 μmol g$^{-1}$ wet weight, although $t_{1/2}$ elimination rates were unchanged. IF was also detected in the liver in vivo, with a $t_{1/2}$ of about 1 h. Carbogen breathing did not affect the maximum peak area ($C_{max}$) or the $t_{1/2}$ in the liver. Most importantly, the carbogen-induced increase in IF uptake by the tumour caused significant growth delay at all time points in the GH3 tumour growth between day 5 and day 12 ($P$ < 0.01) compared with IF alone. These findings show that carbogen breathing has potential for increasing the efficacy of anti-cancer drugs. Isolated GH3 cells were sensitive to the parent drug (IF) in vitro ($IC_{50}$ = 1.3 ± 0.2 mM) suggesting that the GH3 cells may be either expressing P450 enzymes or are sensitive to the parent drug per se.

**Keywords:** ifosfamide; $^{31}$P magnetic resonance spectroscopy; carbogen; chemotherapy; pharmacokinetics

Of the many anti-cancer agents that exist, only a few have been monitored non-invasively by magnetic resonance spectroscopy (MRS) (Malet Martino et al, 1992; Artemov et al, 1995; He et al, 1995). This study was designed to take advantage of the non-invasive technique of $^{31}$P-MRS for pharmacokinetic studies of ifosfamide (IF), an alkylating oxazaphosphorine drug and a structural isomer of cyclophosphamide (Figure 1), which is widely used in the treatment of soft-tissue sarcomas and paediatric malignancies (Pinkerton and Pritchard, 1989; Pratt et al, 1989.) IF is a prodrug that is metabolized in vivo to produce a range of therapeutically active and potentially toxic metabolites (Sladek, 1988), and its initial metabolism consists of two different pathways (Figure 1). Firstly, it can be activated by hepatic cytochrome P450 enzymes (Allen et al, 1976; Clarke and Waxman, 1989) to give 4-hydroxyifosfamide (4-OHIF), which equilibrates with its tautomer, aldoIF, both of which are membrane permeable (Boyd et al, 1980; Sonawat et al, 1990). The latter can be oxidized by aldehyde dehydrogenase (ALDH) to give inactive carboxyifosfamide, a cause of chemoresistance, or it can decompose by β-elimination to give isophosphoramide mustard (IPM), the primary alkylating agent. The IPM has a p$K_a$ of 4.75 and is membrane impermeable (Engle et al, 1979) and therefore has to be formed intracellularly for its cytotoxic action. Secondly, up to 50% of IF can undergo spontaneous oxidative dealkylation to give 2- or 3-dechloroethylifosfamide (2-DCI or 3-DCI) and chloroacetaldehyde, a compound with possible neurotoxic properties.

*Received 28 March 1996*
*Revised 22 August 1996*
*Accepted 28 August 1996*

*Correspondence to:* LM Rodrigues

IF and its metabolites are usually determined by classical techniques (fluorimetry, mass spectrometry, chromatography) on derivatized tissue extracts or body fluids (Wagner et al, 1981; Lind et al, 1989; Boddy et al, 1993). More recently however, $^{31}$P-MRS has also been used in vitro to follow the metabolism of two other

**Figure 1** Structure and metabolism of ifosfamide oxazaphosphorine cytostatics, cyclophosphamide and mafosfamide, in cultured tumour cells (Boyd et al, 1980; Sonawat et al , 1990) and to analyse IF metabolities in body fluids from patients (Martino et al, 1992; Gilard et al, 1993). With MRS, it is possible to perform 'pharmacokinetics in situ' on the drug at its site of action. The primary purpose of the present study was to use $^{31}P$-MRS to monitor the pharmacokinetics of IF in rat tumours in vivo, with simultaneous monitoring of the endogenous phosphorus metabolites (phosphocreatine (PCr), nucleoside triphosphate (NTP), inorganic phosphate ($P_i$)) using a bolus dose previously used in animals (Wiedemann et al, 1993). Doses similar to that used in the present study are administered to patients, but as slow infusions rather than a bolus (as reviewed by Kaijser et al, 1994).

Many tumours have poor vascularity and tend to be resistant to radiotherapy and chemotherapy because of factors ranging from intrinsic genetic resistance to extrinsic physiological factors (as reviewed by Vaupel et al, 1989). As some studies have shown that it is possible to enhance tumour blood flow and/or oxygenation by carbogen (95% oxygen/5% carbon dioxide) breathing in animals (Kruuv et al, 1967; Honess et al, 1995; Robinson et al, 1995) and humans (Falk et al, 1992), the effects of carbogen breathing on IF pharmacokinetics were also studied. Carbogen breathing has also been shown to increase the efficacy of radiotherapy (Rojas 1991). Could the increase in blood flow – oxygenation observed with carbogen breathing (thought to be due partly to vasodilatory effects of carbon dioxide), increase tumour delivery of the prodrug IF, or of its activated metabolites, and thereby enhance chemotherapeutic action? Furthermore, the carbogen-induced increase in tumour blood flow is rapidly reversed when air breathing is resumed (Robinson et al, 1995), and thus it may be possible to trap IF inside the tumour. To evaluate the efficacy of the IF, both with and without carbogen breathing, tumour growth rate was measured.

As activation of IF to 4-OHIF occurs primarily in the liver and carbogen breathing has been shown to increase relative tumour perfusion, but not liver perfusion in RIF-1 tumour-bearing mice (Honess et al, 1995), pharmacokinetic measurements in situ were also performed on the liver with and without carbogen. To assess the possibility that the prodrug IF itself might be directly toxic to rat pituitary GH3 tumour cells, or might be converted by them to a toxic metabolite, its effect on GH3 cells in vitro was also monitored, using IF concentrations similar to those used in vivo.

### METHODS

Ifosfamide (Mitoxana, ASTA Medica) was made up freshly each day in normal saline, pH 7.2–7.4 and administered i.v. at a dose of 250 mg $kg^{-1}$, which is equivalent to 1.5 g $m^{-2}$, using the surface law formula (Benedict, 1934). If the animal was to recover from the anaesthetic, MESNA (Uromitexan), a uroprotector was administered i.v. (150 mg $kg^{-1}$).

### Cell culture

GH3 prolactinoma cells were grown in RPMI 1640 medium with glutamine, supplemented with 10% fetal bovine serum, 5% horse serum and 50 μg $ml^{-1}$ gentamycin at 37°C in a 5% carbon dioxide atmosphere. Cell number was measured either directly using a haemocytometer, or indirectly using 3-[4,5-dimethylthiazol-2-yl]-2,5-diphenyltetrazolium bromide (MTT) in a colorimetric assay. For the MTT assay, cells were seeded at 3300 $cm^{-2}$ in 100-μl aliquots, in quintuplet using 96-well plates. Twenty-four hours later, IF, freshly prepared in Hanks' buffered salt solution (HBSS), was diluted in growth medium and 100 μl added to wells to give final concentrations of 0.3–3 mM. After 24, 48 and 72 h, MTT was added (final concentration 0.45 mg $ml^{-1}$), followed 4 h later by sodium dodecyl sulphate (SDS) (final concentration 5%). After ≥20 h, the plates were read at 540 nm using a microplate reader. Results were expressed as a fraction of control (untreated cultures) after conversion of absorbance readings to cell number, based upon our standard curve for these cells. All cell culture materials were purchased from Gibco BRL (Paisley, UK), apart from the thiazolyl blue dye MTT and the SDS, which were obtained from Sigma (Poole, UK).

### Tumours

The GH3 prolactinomas were grown in the flanks of female Wistar–Furth rats as described previously (Prysor-Jones and Jenkins, 1981). The *N*-methyl-*N*-nitrosourea (MNU) mammary tumours were chemically induced by three injections of MNU at 2-weekly intervals into Ludwig/Wistar/Olac rats, essentially as described by Williams et al (1985). Tumour diameter was measured with callipers, and the volume calculated using the formula $(\pi/6)d_1d_2d_3$.

### $^{31}P$-MRS

Animals were anaesthetized with pentobarbitone (40 mg $kg^{-1}$) and maintained at 37°C. The tail vein was cannulated and an i.v. line placed for administration of IF while the animal remained in the magnet. $^{31}P$-MR spectra were obtained on a 4.7-T SISCO 200/300 spectrometer with a 20-mm, two-turn surface coil. Non-localized spectra of tumours and livers, the latter from non-tumour-bearing rats, were obtained using an adiabatic sincos pulse, TR of 3 s or 5 s and 60 or 120 acquisitions. Image-selected in vivo (ISIS)-localized spectra (Ordidge et al, 1986) were obtained to confirm the presence of IF in the tumour as distinct from the adjacent tissues. However, the ISIS-localization is unsuitable for pharmacokinetic studies because the spectra take too long to acquire. In the carbogen experiments ($n$ = 4), animals breathed carbogen from a mask equipped with a scavenger, at a flow rate of 1.8 l $min^{-1}$ for 10 min. IF was administered during the tenth minute of carbogen breathing. Air was given before and after the carbogen. Mean arterial blood pressure was measured using a Harvard Rat Tail BP Monitor (Harvard Apparatus, Edenbridge, Kent, UK).

### Measurements of pH and quantitation of spectra pH was calculated from the chemical shift of ($P_i$ relative to α-NTP at – 7.57 p.p.m. by the method of Prichard et al (1983). Spectra were quantitated using VARPRO, a time domain non-linear least squares method (van der Veen et al, 1988). To normalize the data, the IF level was expressed as a ratio of IF to total phosphate signals [i.e. α-, β- and γ-NTP, PCr, $P_i$ and phosphomonoester (PME)] in the spectra as total phosphate was unlikely to change over the time course of the experiments.

### Pharmacokinetic analysis

Curve fitting of the data (peak area *vs* time) was achieved using the software package PCNONLIN (Lexington, KY, USA), which provides a least squares estimate of the parameters in a non-linear

© Cancer Research Campaign 1997

**Figure 2** Representative $^{31}$P-MR spectra 15 min after i.v. IF (250 mg kg$^{-1}$) in (**A**) a GH3 prolactinoma and (**B**) an MNU-induced mammary tumour. Peak assignments are as follows: 1, ifosfamide; 2, phosphomonoesters; 3, $P_i$; 4, phosphodiesters; 5, phosphocreatine; 6, γ-phosphate of NTP; 7, α-phosphate of NTP; 8, β-phosphate of NTP

**Table 1** Effect of carbogen breathing on IF pharmacokinetics in the GH3 prolactinoma and normal liver obtained from in vivo $^{31}$P-MR spectra

| Tissue | n | $C_{max}$ | $t_{1/2}$(min) | AUC |
|---|---|---|---|---|
| GH3 tumour | 5 | 0.059 ± 0.002 | 208 ± 25 | 17.9 ± 2.4 |
| GH3 tumour +carbogen | 4 | 0.094 ± 0.006[a] | 215 ± 27 | 28.9 ± 3.7[b] |
| Liver | 4 | 0.063 ± 0.009 | 59 ± 10 | 5.1 ± 0.6 |
| Liver +carbogen | 4 | 0.076 ± 0.002[c] | 71 ± 16 | 7.7 ± 1.6[c] |

Results show the means ± sem, where $C_{max}$ is the maximum peak area expressed as a measure of IF/ΣP, $t_{1/2}$ is half-life for elimination and AUC is the area under (IF/ΣP) *vs* time curve. [a]$P < 0.001$, [b]$P < 0.05$, [c]$P > 0.15$ compared with air breathing.

model. The best fit was obtained using a one-compartment model with bolus input and first-order output using the equation:

$$CT = D/V{*}\exp(-K_{el}{*}T)$$

where $C$ = concentration (peak area), $T$ = time (min), $D$ = dose, $V$ = volume and $K_{el}$ = elimination rate. This yielded values of $C_{max}$ and $t_{1/2}$ (= $0.693/K_{el}$) for each individual tumour.

**Figure 3** Effect of i.v. IF (250 mg kg$^{-1}$) on NTP/$P_1$ (●, ▲) and pH (○, △) in GH3 prolactinomas (●, ○) and mammary tumours (▲, △). Data normalized to pre-IF values of 1.32 ± 0.1 in GH3 prolactinomas and 2.09 ± 0.49 in mammary tumours. Means ± s.e.m. for three or four animals, **$P \leq 0.002$, *$P \leq 0.03$ (Student's *t*-test) compared with pre-IF value

### RESULTS

### Uptake of IF in GH3 and MNU-induced mammary tumours in vivo

The $^{31}$P-MRS spectra of the tumours showed resonances from PME, $P_i$, phosphodiesters, PCr and from the α-, β- and γ-phosphates of NTP, all typical of tumour spectra (Figure 2). In addition, a $^{31}$P-MR resonance, + 18 p.p.m. from PCr, which corresponded to IF, was detected in both the GH3 prolactinoma (Figure 2A) and the MNU-induced mammary tumour (Figure 2B) within a few minutes of injection and was still visible 5–6 h later (not shown in Figure 2). ISIS-localized acquisitions confirmed the presence of this IF signal in the tumour per se (results not shown). By comparing the integral of the IF peak with that of β-NTP, which is known to correspond to about 1.4 μmol g$^{-1}$ wet weight in this tumour type (van den Boogaart et al, 1995), the average concentration of IF in the tumour tissue over the first 2 h was calculated to be 0.42 μmol g$^{-1}$ wet weight. (Corrections were made to account for the small but significant initial decrease in NTP observed after injection of IF – see below.)

Time courses were pursued up to 6 h after injection and the mean half-life of IF ($t_{1/2}$) was calculated to be 208 ± 25 min ($n$ = 5) in the prolactinoma (see also Table 1) and 165 ± 19 min ($n$ = 3) in the mammary tumour (data not shown). The time course showed an initial rapid uptake followed by elimination of the drug over approximately 6 h (all time points not shown in Figure 5). No IF was detectable 24 h later.

### Effect of IF on endogenous tumour phosphorus metabolites

NTP/$P_i$ decreased significantly ($P \leq 0.002$) to between 60% and 85% of the original value in all the tumours within 30 min of IF administration (Figure 3). There was also a concomitant fall in pH of between 0.05–0.15 pH units in all tumours although it was significant ($P < 0.05$) only in the GH3 prolactinomas. Parallel experiments were performed to test the hypothesis that these changes were due to hypotension. In four out of six rats studied the mean arterial blood pressure fell to 70–90% of control within

© Cancer Research Campaign 1997

**Figure 4** Effect of carbogen breathing on the $^{31}$P spectrum of a GH3 prolactinoma. (**A**) IF administered after 10 min breathing air. (**B**) IF administered during tenth minute of breathing carbogen. The spectra were acquired 15 min after i.v. IF. Peak assignments as in Figure 2

**Figure 5** Effect of carbogen breathing on IF pharmacokinetics [(IF/ΣP) *vs* time] in GH3 prolactinomas measured by $^{31}$P-MRS. ○, Control animals; ●, animals breathing carbogen. Means ± s.e.m for three to five animals. $P \leq 0.01$ (Student's *t*-test) for all time points except at 10 min ($P = 0.02$) and 120 min ($P = 0.18$)

10–15 min of 25 mg kg$^{-1}$ i.v. IF, and in three of those four rats it recovered within 2 h (the fourth rat died without recovery of normal blood pressure). While not fully conclusive, these results suggest that the fall in NTP/$P_i$ and pH observed after i.v. bolus IF could be due to drug-induced hypotension. Complete recovery to control levels of both these parameters took between 1 and 2 h.

**Figure 6** Effect of carbogen breathing on IF pharmacokinetics [(IF/ΣP) *vs* time] in normal liver measured by $^{31}$P-MRS. □, Control animals; ■, animals breathing carbogen. Means ± s.e.m for three to four animals ($P > 0.05$, Student's *t*-test)

### Effects of carbogen breathing on IF pharmacokinetics in GH3 prolactinomas

Carbogen breathing by the rats bearing GH3 prolactinomas markedly increased IF uptake compared with controls breathing air. Within 10 min of drug administration, the IF peak in the tumour spectra after carbogen breathing (Figure 4B) was significantly higher ($P < 0.05$) than in control IF-treated rats (Fig 4A). As before, by comparing the integral of the IF peak with that of β-NTP, the average concentration of IF in the tumour tissue over the first 2 h (not shown in Figure 4) was calculated to be about 0.68 μmol g$^{-1}$ wet weight after carbogen breathing (compared with the air-breathing value of 0.42 μmol g$^{-1}$ wet weight). The differences were maintained throughout the time course (see Figure 5) and were still significant at 150 min ($P < 0.01$). There was also a significant increase in the area under the (IF/ΣP) *vs* time curve (AUC) ($P < 0.05$) with carbogen breathing. However, the average increase of about 50% in the initial IF concentration and in the AUC was not accompanied by a significant change in the $t_{1/2}$ for elimination (Table 1).

In rat liver, IF was also detected in vivo within a few minutes of injection (spectra not shown) with a $t_{1/2}$ for elimination of 59 ± 10 min. However, carbogen breathing did not significantly affect the pharmacokinetics of IF in the liver (Figure 6). There were no significant differences either in $t_{1/2}$ elimination rate (71 ± 16 min) or in $C_{max}$ in contrast to the significant difference in $C_{max}$ in the tumour (see Table 1).

### Effects of carbogen breathing on the efficacy of IF assessed by measurements of GH3 tumour growth

IF alone caused significant growth delay compared with untreated tumours at day 12 ($P < 0.02$). Carbogen breathing in addition to IF caused a further significant delay at all time points in GH3 tumour growth between day 5 ($P < 0.05$) and day 12 ($P < 0.01$) compared with the rats treated with IF alone (Figure 7). At day 13, untreated tumours were 655 ± 75% of the size on day 0, whereas tumours of the IF-treated rats, which had also breathed carbogen, showed negligible growth 12 days after treatment.

© Cancer Research Campaign 1997

**Figure 7** Effect of IF and carbogen breathing on GH3 tumour volumes. ○, Control animals; ●, after i.v. IF (250 mg $kg^{-1}$); △, after carbogen breathing; and ▲, after IF plus carbogen breathing. Means ± s.e.m. Volume of tumours at start of treatment was 2.89 ± 0.3 $cm^3$ ($n$ = 12)

**Figure 8.** Effect of different concentrations of IF on GH3 cell proliferation in vitro. After 24 h incubation of the cells, IF was either absent (■) or added to a final concentration of 0.3 mM (●), 1 mM (▲) or 3 mM (▼). The absorbance of the cell suspension was determined at 24-h intervals and converted to cell number (for details see Methods). Results are expressed as means ± s.e.m. of three experiments using normalized data. *$P$<0.01 (Fisher's ANOVA)

### Metabolites of IF

No active metabolites of IF were seen in any of the in vivo tumour spectra, although some in vivo liver spectra showed the presence of carboxyifosfamide (data not shown).

### Effect of IF on cultured GH3 cells

In an initial study using simple cell counting, incubation of IF (3 mM) with exponentially growing GH3 cells caused a significant decrease in cell number at 24, 48 and 72 h. All subsequent studies used the more sensitive MTT assay. Maximum growth inhibition by IF was seen at 3 mM, whereas 0.3 mM had no significant effect (Figure 8). The concentration of 3 mM reproducibly inhibited cell proliferation yielding an $IC_{50}$ at 72 h of 1.3 ± 0.2 mM (mean±s.e.m., $n$=3). To test the possibility that the cytotoxicity of IF was due to chemical breakdown in the medium, IF was preincubated in cell culture medium (37°C) or in HBSS (4°C) for 48 h before addition to the cells at a final concentration of 3 mM for 24 h. The percentage inhibition of growth observed for these two treatments of 25.8±0.3% and 29.2±0.5%, respectively, was actually less than that in which IF was added immediately to the cells (44.4 ± 0.3%), demonstrating that cell-independent breakdown of IF to cytotoxic metabolites was not significant.

### DISCUSSION

Monitoring IF by $^{31}$P-MRS has several significant advantages over chromatographic methods. It enables one to monitor the fate of a drug in a living system without prior treatment, thus avoiding problems encountered with sampling, extraction, recovery and derivatization. Furthermore, a complete time course can be followed in the tumour of a single animal. The application of MR spectroscopy to IF metabolism has so far been reported in vitro, in studies of body fluids (Martino et al, 1992; Gilard et al, 1993) and isolated cells (Boyd et al, 1980; Sonowat et al, 1990), with one in vivo study (Krems et al, 1994). In this study, we have shown that $^{31}$P-MRS can be used to monitor the pharmacokinetics of IF with simultaneous observation of the endogenous phosphorus metabolites, without the need for serial biopsies. This has provided information on the distribution and metabolism of the drug, while MRS in vitro has given information on its excretion and metabolism. However, the main limitation of $^{31}$P-MRS is its low sensitivity compared with chromatographic methods. Indeed, no IF metabolites were detected in vivo or in extracts of the two tumour types, probably because the levels were below the detection threshold (about 0.2 mM in vivo and 20 μM in vitro depending on magnetic field strength). 4-OHIF, the activated form of IF, is extremely labile and difficult to measure even by chromatographic techniques. Although carboxyifosfamide (an inactive metabolite) was detected in some of the livers, it was not detected in any tumours. There is some evidence that the presence of carboxy ifosfamide in tumours might indicate chemoresistance to IF (Boal et al, 1994).

The intratumoral pharmacokinetics of IF were reproducibly measured in both types of tumour and in the liver. While host liver metabolism of IF is an important determinant of tumour response, metabolism by the tumour itself may also be important in some instances. Previous cultured-cell studies have used the activated form of IF, 4-OHIF, as activation of IF occurs predominantly in the liver. However, cytochrome P450 expression has recently been shown in malignant breast cancer cells (Murray et al, 1993), and therefore it is possible that activation could take place in the cancer cell. Our results using isolated GH3 cells showed cytotoxicity of the parent drug (IF) in vitro, suggesting that the GH3 prolactinoma cells may be either (1) expressing the P450 enzymes necessary for the initial activation of IF or (2) sensitive to the parent drug per se. The maximum IF concentration observed in tumours with carbogen breathing in vivo [on the assumption that the area under the β-NTP is equivalent to 1.4 μmol $g^{-1}$ wet weight (van den Boogaart et al, 1995)] was 0.82 μmol $g^{-1}$ wet weight at 15 min after IF treatment, similar in magnitude to the $IC_{50}$ found in the cell culture experi-

 © Cancer Research Campaign 1997

ments (1.3 mM). Furthermore, the concentration–response curve in vitro was steep, with 0.3 mM having no effect. This suggests that the 50% increase in tumour IF induced by carbogen breathing could be biologically very significant.

The rationale for using carbogen inhalation to modify IF pharmacokinetics and pharmacodynamics was also based on observations suggesting that the action of this prodrug could be enhanced by the improvement in tumour blood flow induced by hyperthermia, both in cancer patients (Issels et al, 1990) and in human tumour xenografts (Wiedemann et al, 1993). Wiedemann et al showed that, with rising tumour temperature, the therapeutic efficacy of IF increased more steeply in vivo than in vitro and inferred that this was due to an increase in tumour blood flow with hyperthermia. Carbon dioxide is a powerful vasodilator, hence carbogen inhalation could mimic this effect by increasing blood flow to the poorly perfused regions of the tumour (Honess et al, 1995), simultaneously improving tissue oxygen tension. This effect has been demonstrated by large increases in the intensity of gradient recalled echo magnetic resonance images (GRE MRI) of GH3 prolactinomas (Robinson et al, 1995), consistent with an increase in both tumour blood flow and oxygenation, which return rapidly to normal when carbogen is replaced by air breathing. Here, we have shown by in vivo $^{31}$P-MRS that carbogen breathing increases the concentration of the prodrug, IF, within the tumour. This is probably due to an increase in blood flow, and it is possible that it may be accompanied by an increased delivery of the activated form of IF, 4-hydroxyifosfamide. Alternatively, this tumour may be able to activate IF in situ, as suggested by the in vitro studies, but whatever the mechanism, more IF is taken up and retained by the tumour (relative to that taken up by tumours of air-breathing animals), and the chemotherapeutic index is increased.

The increase in the tumour concentration of IF with carbogen breathing was not mirrored by an increase in liver IF. Our preliminary results show no evidence of GRE MRI signal intensity increases in the liver, and therefore this was not unexpected. This is also in agreement with the findings of Honess et al (1995), who showed an increase in tumour, lung and skin perfusion caused by carbogen breathing but no effect in normal liver, kidney, skeletal muscle and spleen. Whether carbogen breathing causes increased uptake of drug in the central nervous system and kidney, sites of unwanted cytotoxity in patients (Goren et al, 1986 and Skinner et al, 1990), is not known.

The ability to monitor in vivo the fate of a drug in a living system may allow us to explore the fundamental mechanisms underlying drug action as well as pharmacokinetic parameters that could provide useful information on response to chemotherapy. We have recently demonstrated, using GRE MRI, that carbogen inhalation increases tumour blood flow and oxygenation in patients (Taylor et al, 1996). The current study also suggests that breathing carbogen may be useful in the clinic for selectively increasing the uptake of chemotherapeutic agents into tumours, thereby minimizing normal tissue toxicity and enhancing the efficacy of treatment.

### ACKNOWLEDGEMENTS

This work was supported by a Cancer Research Campaign programme grant (SP1971/0402) and project grant (SP1971/0501). We thank Rick Skilton and his staff for maintenance of the livestock.

### REFERENCES

Allen LM, Creavan PJ and Nelson RL (1976) Studies on the human pharmacokinetics of isophosphamide (NSC 109724) *Cancer Treat Rep* **60**: 451–458

Artemov D, Bhujwalla ZM, Maxwell RJ, Griffiths JR, Judson IR, Leach MO and Glickson JD (1995) Pharmacokinetics of the $^{13}$C labelled anticancer agent temozolomide detected *in vivo* by selective cross polarization transfer. *Magn Reson Med*, **34**, 338–342

Benedict FG (1934) Die oberflachen bestimmung verschiedener tiergattungen. *Ergeg Physiol*, **36**, 300–346

Boddy AV, Yule SM, Wyllie R, Price L, Pearson ADJ and Idle JR (1993) Pharmacokinetics and metabolism of ifosfamide administered as a continuous infusion in children. *Cancer Res* **53**, 3758–3764

Boal JH, Ludeman SM, HO C-K, Engel J and Niemeyer U (1994) Direct detection of the intracellular formation of carboxyphosphamides using nuclear magnetic resonance spectroscopy. *Arzneim-Forsch/Drug Res* **44** (1): 84–93

Boyd VL, Robbins JD, Egen W and Ludeman SM (1980) $^{31}$P NMR spectroscopic observation of the intracellular transformation of oncostatic cyclophosphamide metabolites. *J Med Chem* **29**, 1206–1210

Clarke L and Waxman DJ (1989) Oxidative metabolism of cyclophosphamide: identification of the hepatic monooxygenase catalyst of drug activation. *Cancer Res* **50**, 4991–5002

Engle TW, Zon G and Egan W (1979) $^{31}$P NMR investigations of phosphoramide mustard: evaluation of pH control over the rate of intramolecular cyclization to an aziridinium ion and the hydrolysis of this reactive alkylator. *J Med Chem* **22**, 897–899

Falk SJ, Ward R and Bleehen NM (1992) The influence of carbogen breathing on tumour tissue oxygenation in man evaluated by computerised $pO_2$ histography. *Br J Cancer* **66**, 919–924

Gilard V, Malet-Martino MC, de Forni M, Niemeyer U, Ader JC and Martino R (1993) Determination of the urinary excretion of ifosfamide and its phosphorated metabolites by phosphorous-31 nuclear magnetic resonance spectroscopy. *Cancer Chemother Pharmacol* **31**, 387–394

Goren MP, Wright RK, Pratt CB and Pell FE (1986) Dechloroethylation of ifosfamide and neurotoxicity. *Lancet* **2**, 1219–1220

HE Q, Bhujwalla ZM, Maxwell RJ, Griffiths JR and Glickson JD (1995) Proton NMR observation of the antineoplastic agent iproplatin *in vivo* by selective multiple quantum coherence transfer (Sel-MQC). *Magn Reson Med* **33**, 414–416

Honess DJ and Bleehen NM (1995) Perfusion changes in the RIF-1 tumour and normal tissues after carbogen and nicotinamide, individually and combined. *Br J Cancer* **72**, 1175–1180

Issels RD, Prenninger SW, Nagele A, Boehm E, Sauer H, Jauch KW, Denecke H, Berger H, Peter K and Wilmanns W (1990) Ifosfamide plus etoposide combined with regional hyperthermia in patients with locally advanced sarcomas: a phase II study. *J Clin Oncol* **8**, 1818–1829

Kaijser GP, Beijnen JH, Bult A and Underberg WJM (1994) Ifosfamide metabolism and pharmacokinetics (Review). *AntiCancer Res* **14**, 517–532

Krems B, Bachert P, Haberkorn U, Gerlach L, Wiessler M, Pohl J, van Kaick G and Lorenz WJ (1994) Non-invasive pharmacokinetic measurement of glucosyl-ifosfamide in tumour bearing rats by means of *in vivo* $^{31}$P NMR spectroscopy. *Proc Soc Magn Reson* 2nd Meeting, 1317

Kruuv JA, Inch WR and McCredie JA (1967) Blood flow and oxygenation of tumours in mice. I. Effects of breathing gases containing $CO_2$ at atmospheric pressure. *Cancer* **20**, 51–58

Lind MJ, Margison JM, Cerny T, Thatcher N and Wilkinson PM (1989) Comparitive pharmacokinetics and alkylating activity of fractionated intravenous and oral IF in patients with bronchogenic carcinoma. *Cancer Res* **49**, 753–759

Malet-Martino MC and Martino R (1992) Magnetic Resonance Spectroscopy: a powerful tool for drug metabolic studies. *Biochimie* **74**, 785–800

Martino R, Crasnier F, Chouini-Lalanne N, Gilard V, Niemeyer U, de Forni M and Malet-Martino MC (1992) A new approach to the study of ifosfamide metabolism by the analysis of human body fluids with $^{31}$P nuclear magnetic resonance spectroscopy. *J Pharmacol Exp Ther* **260**, 1133–1144

Murray GI, Weaver RJ, Paterson PJ, Ewem SWB, Melvin WT and Burke D (1993) Expression of xenobiotic metabolizing enzymes in breast cancer. *J Pathol* **169**, 347–353

Ordidge RA, Connelly A and Lohman JAB (1986) Image-selected *in vivo* spectroscopy (ISIS). A new technique for spatially selective NMR spectroscopy. *J Magn Reson* **66**, 283–294

Pinkerton CR and Pritchard J (1989) A phase II study of ifosfamide in paediatric tumours. *Cancer Chemother Pharmacol* **24** (suppl.): 13–15

© Cancer Research Campaign 1997

Pratt CB, Douglass EC and Etcubanas EL (1989) Ifosfamide in paediatric malignant solid tumours. *Cancer Chemother Pharmacol* **24** (suppl.): 24–27

Prichard JW, Alger JR, Behar KL, Petroff OA and Shulman RG (1983) Cerebral metabolic studies *in vivo* $^{31}$P NMR. *Proc Natl Acad Sci USA* **80**, 2748–2751

Prysor-Jones RA and Jenkins JS (1981) Effect of bromocriptine on DNA synthesis, growth and hormone secretion of spontaneous pituitary tumours in rat. *J Endocrinol* **88**, 463–469

Robinson SP, Howe FA and Griffiths JR (1995) Noninvasive monitoring of carbogen-induced changes in tumour blood flow and oxygenation by functional magnetic resonance imaging. *Int J Radiat Oncol Biol Phys* **32**, 855–859

Rojas A (1991) Radiosensitization with normobaric oxygen and carbogen. *Radioth Oncol* **20** (suppl.): 65–70

Skinner R., Pearson ADJ., Price L., Coulthard MG and Craft AW (1990) Nephrotoxicity after ifosfamide. *Arch Dis Child* **65**, 732–738

Sladek N (1988) Metabolism of oxazaphosphorines. *Pharmacol Ther* **37**, 301–355

Sonowat HM, Leibfritz D, Engel J and Hilgard P (1990) Biotransformation of mafosfamide in P388 mice leukemia cells: intracellular $^{31}$P MR studies. *Biochiem Biophys Acta* **1052**, 36–41

Taylor NJ, Griffiths JR, Howe FA, Saunders MI, Robinson SP, Thoumine M, Caine LA, Hoskin PJ, Powell M, Chaplin DJ and Baddeley H (1996) Carbogen-induced oxygenation and blood flow changes within human tumours, monitored by Gradient Echo Magnetic Resonance Imaging. *Proc Int Soc Magn Reson Med* **2**:1313

van den Boogaart A, Howe FA, Rodrigues LM, Stubbs M and Griffiths JR (1995) *In vivo* $^{31}$P MRS: absolute concentrations, signal to noise and prior knowledge. *NMR Biomed* **8**, 87–93

van der Veen JWC, de Beer R, Luyten PR and van Ormondt D (1988) Accurate quantification of *in vivo* $^{31}$P NMR signals using the varible projection method and prior knowledge. *Magn Reson Med* **6**, 92–98

Vaupel P, Kallinowski F and Okunieff P (1989) Blood flow, oxygen and nutrient supply, and metabolic microenvironment of human tumours: a review. *Cancer Res* **49**, 6449–6465

Wagner T, Heydrich D, Jork T, Voelcher G and Hohorst HJ (1981) Comparitive study of activated ifosfamide and cyclophosphamide by a modified fluorometric test. *J Cancer Res Clin Oncol* **100**, 95–104

Wiedemann GJ, Siemens HJ, Mentzel A, Biersack A, Wossmann W, Knocks D, Weiss C and Wagner T (1993) Effects of temperature on the therapeutic efficacy and pharmacokinetics of ifosfamide. *Cancer Res* **53**, 4268–4272

Williams JC, Gusterson B, Humphreys J, Monaghan P, Coombes RC and Rudland PS (1985) Isolation and characterisation of clonal cell lines from a transplantable metastasizing rat mammary tumour, TR2CL. *J Natl Cancer Inst* **74**: 415–428

© Cancer Research Campaign 1997